# Genome sequence of *Candidatus* Arsenophonus lipopteni, the exclusive symbiont of a blood sucking fly *Lipoptena cervi* (Diptera: Hippoboscidae)

**DOI:** 10.1186/s40793-016-0195-1

**Published:** 2016-09-17

**Authors:** Eva Nováková, Václav Hypša, Petr Nguyen, Filip Husník, Alistair C. Darby

**Affiliations:** 1 Faculty of Science, University of South Bohemia, and Institute of Parasitology, Biology Centre, ASCR, v.v.i. Branisovka 31, 37005 Ceske Budejovice, Czech Republic; 2 Faculty of Science, University of South Bohemia, and Institute of Entomology, Biology Centre, ASCR, v.v.i. Branisovka 31, 37005 Ceske Budejovice, Czech Republic; 3 Institute of Integrative Biology, University of Liverpool, Crown Street, Liverpool, L69 7ZB UK

**Keywords:** *Arsenophonus*, Symbiosis, Tsetse, Hippoboscidae

## Abstract

*Candidatus* Arsenophonus lipopteni (*Enterobacteriaceae*, *Gammaproteobacteria*) is an obligate intracellular symbiont of the blood feeding deer ked, *Lipoptena cervi* (Diptera: Hippoboscidae). The bacteria reside in specialized cells derived from host gut epithelia (bacteriocytes) forming a compact symbiotic organ (bacteriome). Compared to the closely related complex symbiotic system in the sheep ked, involving four bacterial species, *Lipoptena cervi* appears to maintain its symbiosis exclusively with *Ca. Arsenophonus lipopteni*. The genome of 836,724 bp and 24.8 % GC content codes for 667 predicted functional genes and bears the common characteristics of sequence economization coupled with obligate host-dependent lifestyle, e.g. reduced number of RNA genes along with the rRNA operon split, and strongly reduced metabolic capacity. Particularly, biosynthetic capacity for B vitamins possibly supplementing the host diet is highly compromised in *Ca. Arsenophonus lipopteni*. The gene sets are complete only for riboflavin (B2), pyridoxine (B6) and biotin (B7) implying the content of some B vitamins, e.g. thiamin, in the deer blood might be sufficient for the insect metabolic needs. The phylogenetic position within the spectrum of known *Arsenophonus* genomes and fundamental genomic features of *Ca. Arsenophonus lipopteni* indicate the obligate character of this symbiosis and its independent origin within Hippoboscidae.

## Introduction

Symbiosis has for long been recognized as one of the crucial drivers of evolution. In insects, numerous symbiotic relationships, mainly with bacteria, enabled the hosts to exploit various environments and/or life strategies, and supposedly started adaptive radiations in some groups. The mechanisms of such evolutionary processes include for example contribution to the host immunity, modification of the reproductive strategy, or provision of essential compounds to the hosts relying on nutritionally compromised resources. Blood feeding (hematophagous) insects provide an illuminating example of a life strategy shift coupled with symbiosis. Since blood meal lacks some of the B vitamins, hematophagous insects rely on their supply by symbiotic bacteria. The relationships between bacteria and hematophagous insect displays considerable degree of variability spanning from less intimate associations with entire gut microbial community, e.g. triatomine bugs [1, 2], to highly specialized interactions with few or single obligate symbiont(s), e.g. lice, bed bugs, tsetse flies, louse flies and bat flies [3–7]. With the recent advancement of genomic approaches and genetic manipulations, symbioses in these insect groups, often important disease vectors, have become of a high interest.

Here we describe fundamental biological characteristics and genome properties of the obligate symbiont of a deer ked, *Lipoptena cervi* (Hippoboscidae). In comparison to multipartite symbiotic systems of closely related hosts from families Hippoboscidae (i.e. *Melophagus ovinus* [7]) and Glossinidae (i.e. *Glossina* sp. [6]), *Lipoptena cervi* harbors a single unaccompanied obligate symbiont from the genus *Arsenophonus*. The genome of *Candidatus* Arsenophonus lipopteni has been sequenced for two reasons. The first was to extend our knowledge on occurrence and genomics of the obligate symbionts across the spectrum of hematophagous hosts involved in strictly bilateral symbiosis, e.g. bed bugs [5], head lice [4], leaches [8, 9]. This is a necessary prerequisite for the future analysis of the origins and evolution of this kind of symbioses. In addition, we intend to use the sequence in a broader comparative framework focused on evolution of bacterial symbiosis, particularly on its role in B vitamin provision to various ecological types of the hosts.

## Organism information

### Classification and features

*Ca*. *Arsenophonus lipopteni* has an obligate association with its host, *L. cervi*, and is therefore uncultivable. In order to localize the bacteria within the host, Fluorescent In Situ Hybridization and Transmission Electron Microscopy was performed on dissected gut tissue as described in detail in [7]. For FISH, the tissue was fixed and hybridized in tubes with eubacterial (EUB338, Flc-GCTGCCTCCCGTAGGA; [10]) and *Ca*. *Arsenophonus lipopteni* specific probes (ArL, Cy3-CTGACTAACGCTTGCACC; this study). The later was designed in a variable region of 16S rRNA gene taking the target sequence accessibility into account [11].

**Fig. 1:**
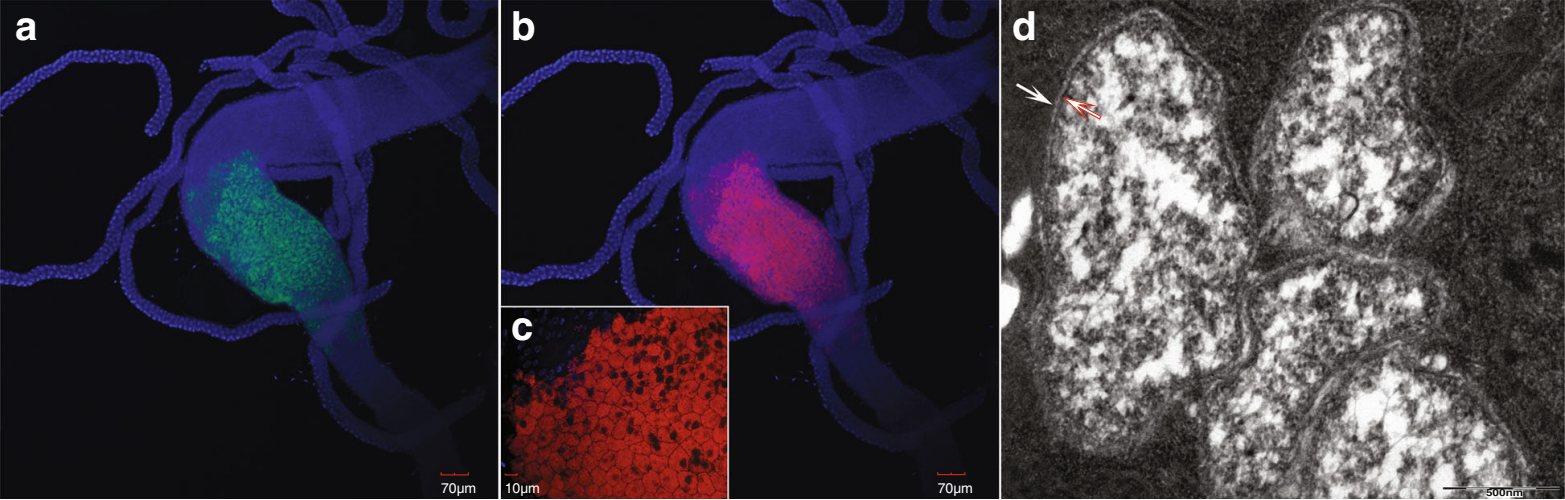
Visualization of the bacteria in the host tissue using FISH and TEM (D). The symbiotic organ (bacteriome) localized in the midgut section harboring *Ca. Arsenophonus lipopteni* targeted with green (Flc) labeled eubacterial probe (**a**) and red (Cy3) labeled specific probe (**b**). Detail of the host cells (bacteriocytes) filled with the symbionts (**c**). The blue signal is DAPI stained DNA. Four cells of *Ca. Arsenophonus lipopteni* under TEM (**d**). The white arrow points to bacterial outer membrane and the red bordered arrow shows the cytoplasmatic cell membrane

The distribution of *Ca*. *Arsenophonus lipopteni* (Fig. 1) in the host body closely resembles that of *Ca*. *Arsenophonus* melophagi and *Wigglesworthia glossinidia*^T^*,* the obligate symbionts of the blood sucking flies *Melophagus ovinus* and *Glossina* sp., respectively [7]. Highly pleomorphic cells of the Gram negative non-sporulating bacteria from the family *Enterobacteriales* are primarily found in the modified part of the gut wall (bacteriome) formed by the specialized enlarged epithelial cells (bacteriocytes, Fig. 1c, 1d). Additional key features of *Ca*. *Arsenophonus lipopteni* are provided as a standardized summary in Table 1.

**Table 1:** Classification and general features of *Ca.* Arsenophpnus lipopteni

MIGS ID	Property	Term	Evidence code^a^	
	Classification	Domain *Bacteria*	TAS	[33]
		Phylum *Proteobacteria*	TAS	[34]
		Class *Gammaproteobacteria*	TAS	[35]
		Order “*Enterobacteriales*”	TAS	[36]
		Family *Enterobacteriaceae*	TAS	[37]
		Genus *Arsenophonus*	TAS	[38]
		Species *Ca. Arsenophonus lipopteni*	IDA	
		Strain: CB	IDA	
	Gram stain	Negative	TAS	[38]
	Cell shape	Pleomorphic	NAS	
	Motility	Non-motile	TAS	[38]
	Sporulation	Non-sporulating	TAS	[38]
	Temperature range	Not determined	IDA	
	Optimum temperature	Not determined	IDA	
	pH range; Optimum	Not determined	IDA	
	Carbon source	Not determined	IDA	
MIGS-6	Habitat	Insect host; bacteriome of *L. cervi*	IDA	
MIGS-6.3	Salinity	Not determined	IDA	
MIGS-22	Oxygen requirement	Not determined	IDA	
MIGS-15	Biotic relationship	Symbiotic	IDA	
MIGS-14	Pathogenicity	Non-pathogen	NAS	
MIGS-4	Geographic location	Ceske Budejovice, Czech Republic	IDA	
MIGS-5	Sample collection date	June 2010	IDA	
MIGS-4.1	Longitude	14.43	IDA	
MIGS-4.2	Latitude	48.97	IDA	
MIGS-4.4	Altitude	399 m	IDA	

^a^Evidence codes, IDA Inferred from Direct Assay, TAS Traceable Author Statement (i.e., a direct report exists in the literature), NAS Non-traceable Author Statement (i.e., not directly observed for the living, isolated sample, but based on a generally accepted property for the species, or anecdotal evidence). These evidence codes are from the Gene Ontology project [39]

Apart from the functional characterization, the genome sequence of Ca. *Arsenophonus lipopteni* was also utilized to assess the relationship of this bacterium to other *Arsenophonus* symbionts. Since the sequence compositional shift compromises phylogenetic usage of 16S rDNA, leading to topological artifacts with long branched symbiotic taxa clustering together [12], we carried out a phylogenetic analysis of a multi-gene matrix and used advanced Bayesian approaches. The matrix was generated for all available *Arsenophonus* genomes (incl. *Ca.**Riesia pediculicola*), five other symbionts, eight non-symbiotic members of *Enterobacteriaceae*, and two outgroups. A set of 70 orthologous genes was determined as an intersection of COGs shared by these bacteria (generated using the MicrobesOnline database; [13]) with “SICO” gene list [14]. The genes were retrieved from the finished assembly using Blastp searches [15] and processed as described previously [7]. The resulting matrix contained 22618 unequivocally aligned positions. Phylobayes [16], a tool specifically developed to overcome the difficulty with heterogeneous composition of sequences, was used for the tree reconstruction. The analysis was run in 2 chains under the GTR + CAT model with amino acids recoded according to the Dayhoff6 option. When the convergence was not reached after 20,000 cycles, the program was stopped and majority rule consensus was calculated after discarding 4,000 cycles burn-in.

**Fig. 2:**
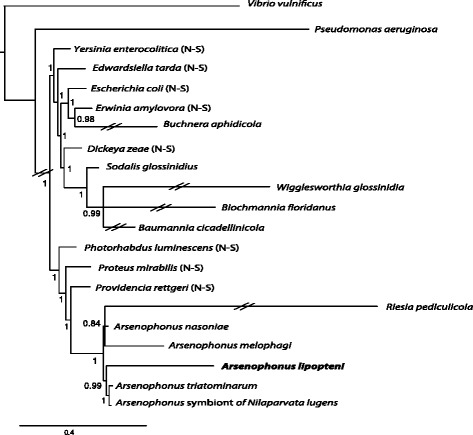
Phylogenomic reconstruction of *Ca. Arsenophonus lipopteni* position. The length of the double crossed branches was scaled to 1:4. The numbers indicate posterior probability for each node. The “N-S” in the brackets following the taxon name designates the non-symbiotic bacteria included into the dataset

The results confirm *Ca*. *Arsenophonus lipopteni* membership in the genus *Arsenophonus*. All *Arsenophonus* species (including *Ca.* Rieisa pediculicola) formed a well-supported monophyletic branch clustering as a sister group to *Providencia* (Fig. 2). Despite the length of branches for the obligate symbionts with highly modified genomes, this arrangement was assigned a high posterior probability. Although the six included *Arsenophonus* lineages certainly do not form a monophyletic group within the known *Arsenophonus* spectrum [17], the results indicate that *Ca.**Arsenophonus lipopteni* evolved independently from *Ca.**Arsenophonus* melophagi housed in related Hippoboscidae host [7].

## Genome sequencing information

### Genome project history

The host specimens *Lipoptena cervi* were collected from wild populations during summer 2010 in the Czech Republic. Finished genome sequence has been deposited in GenBank under acc. No. CP013920 on January 11, 2016. A summary on the sequencing project is provided in Table 2.

**Table 2:** Project information

MIGS ID	Property	Term
MIGS 31	Finishing quality	Finished
MIGS-28	Libraries used	2 × 100 bp paired end
MIGS 29	Sequencing platforms	Illumina
MIGS 31.2	Fold coverage	40
MIGS 30	Assemblers	A5
MIGS 32	Gene calling method	RAST, PGAP, PROKKA
	Locus Tag	AUT07
	Genbank ID	CP013920
	Genbank Date of Release	1-25-2016
	GOLD ID	Gp0127464
	BIOPROJECT	PRJNA306001
MIGS 13	Source Material Identifier	Host tissue
	Project relevance	Evolution of bacterial symbiosis

### Growth conditions and genomic DNA preparation

Since the bacterium is uncultivable, the host tissue was used for DNA extraction. The gut tissue containing the symbiotic organs were dissected from 6 flies in 1× phosphate buffered saline, homogenized with a sterile mortar and pestle and extracted using QiaAmp DNA Micro Kit (QIAGEN, United Kingdom). The DNA quality was assessed using Agilent 2100 Bioanalyzer (Agilent Technologies).

### Genome sequencing and assembly

The paired end 100 bp long reads were generated on one lane of Illumina HiSeq2000 run at Yale Center for Genome Analysis. A5 assembly pipeline with the default settings was used to assemble the reads [18]. Of the 109,640 resulting contigs, the longest contig (836,730 bp) with 40× fold coverage formed a circular molecule with 99 bp overlap at the ends. This contig corresponds to the *Ca.**Arsenophonus lipopteni* genome. Pilon v1.12 [19] was used to check assembly quality and to improve base calls and small indels.

### Genome annotation

The finished genome was annotated using a combination of following tools: RAST [20], PGAP, and Prokka v1.10 [21]. The annotation was then manually curated and checked for the presence of gene remnants. The final annotation is available in GenBank (CP013920). Metabolic pathways were reconstructed in the RAST server [20] and gene absence was verified using BlastP searches. Proteins were assigned to the clusters of orthologous groups using COGnitor [22], and the presence of signal peptides was detected using SignalP [23]. Pfam domains were predicted using HMMER [24] against the Pfam-A database [25]. Transmembrane predictions were done using TMHMM Server v. 2.0. The search for CRISPR repeats was performed in Geneious [26].

## Genome properties

The finished genome consists of 836,724 nucleotides in a single circular chomosome with a low GC content of 24.9 %. The total number of predicted functional genes (667) relative to the genome size implies a lower coding density (75.8 %). The average gene length of 1,001 bp however does not suggest that the genome underwent an extreme economization typical for the obligate symbionts, e.g. *Buchnera aphidicola* str. Cc, *Ca.**Sulcia muelleri*, *Ca.**Carsonella ruddii*, *Ca.**Zinderia insecticolla* [27]. Over 99 % of protein coding genes have been assigned to particular COGs and Pfam domains (Tables 3 and 4). Signal peptides and transmembrane helices have been identified for 5 and 124 protein coding genes respectively (Table 3). The noncoding RNA genes consist of tmRNA, RNAseP, Alpha RBS, cspA, 35 tRNAs, and 3 rRNA genes (altogether 42 RNA genes). The three ribosomal genes are however not organized into a single operon, a phenomenon previously described for at least 9 unrelated bacterial clades, including gammaproteobacterial symbionts of the genus *Buchnera* and *Candidatus* Blochmannia, and attributed to their host-dependent lifestyle [28].

**Table 3:** Statistics for finished genome assebly of *Ca. Arsenophonus lipopteni*

Attribute	Value	% of Total^a^
Genome size (bp)	836,724	100.00
DNA coding (bp)	633,822	75.80
DNA G + C (bp)	208,103	24.90
DNA scaffolds	1	100.00
Total genes	683	100.00
Protein coding genes	625	91.50
RNA genes	42	6.10
Pseudo genes	16	2.30
Genes assigned to COGs	622	99.52
Genes assigned Pfam domains	625	100.00
Genes with signal peptides	5	0.80
Genes with transmembrane helices	124	19.8.00
CRISPR repeats	0	0.00

^a^The total is based on either the size of the genome in bp or the total number of genes

**Table 4:** Number of protein coding genes assigned to the COG categories

Cat. code	Value	Percentage of total	Description
J	128	20.48	Translation, ribosomal structure and biogenesis
A	1	0.16	Processing and modification
K	17	2.72	Transcription
L	40	6.4	Replication, recombination and repair
B	0	0	Chromatin structure and dynamics
D	15	2.4	Cell cycle control, cell division, chromosome partitioning
Y	0	0	Nuclear structure
V	7	1.12	Defense mechanisms
T	10	1.6	Signal transduction mechanisms
M	70	11.2	Cell wall/membrane biogenesis
N	1	0.16	Cell motility
Z	0	0	Cytoskeleton
W	0	0	Extracellular structures
U	12	1.92	Intracellular trafficking and secretion
O	42	6.72	Posttranslational modification, protein turnover, chaperones
C	22	3.52	Energy production and conversion
G	26	4.16	Carbohydrate transport and metabolism
E	34	5.44	Aminoacid transport and metabolism
F	24	3.84	Nucleotide transport and metabolism
H	49	7.84	Coenzyme transport and metabolism
I	26	4.16	Lipid transport and metabolism
P	24	3.84	Inorganic ion transport and metabolism
Q	1	0.16	Secondary metabolites biosynthesis, transport and catabolism
R	9	1.44	General function prediction only
S	13	2.08	Function unknown
-	51	8.16	Assigned to more than one category
-	3	0.48	Not in COGs

The genome properties described above coupled with 16 pseudogenes identified in the genome suggest rather recent establishment of the obligate symbiosis resulting in significant but recent gene/function loss without removal of presently non-coding regions. Regarding the coding capacity for B vitamins and related cofactors, the genome of *Ca.**Arsenophonus lipopteni* appears to be highly economized. Similar to *Ca.**Arsenophonus* melophagi, the bacteria cannot synthesize thiamine (B1), niacin (B3), panthothenic acid (B5) and folic acid (B9). In addition, the genome does not code for heme biosynthesis. Other basic genome characteristics are summarized in Table 3.

## Conclusions

Compared to the closely related complex symbiotic system in the sheep ked, *Melophagus ovinus*, *Lipoptena cervi* appears to maintain symbiosis exclusively with *Ca.**Arsenophonus lipopteni*. The growing number of genome sequences available for the symbionts and the hematophagous hosts involved in strictly bilateral symbiosis (e.g. [29, 30]) will help elucidating some common requirements on B vitamins, or possibly highlight diverse needs of insects digesting blood of various vertebrates. *Ca.**Arsenophonus lipopteni* possesses complete gene sets for biosynthesis of three B vitamins, riboflavin (B2), pyridoxine (B6) and biotin (B7). While the metabolic capacity is directly assessed from genomic data, the presence of any vitamin efflux systems cannot be easily elucidated due to yet poorly understood mechanisms for vitamin export [31]. However, based on recent findings from other hematophagous systems, it has become more clear that the nutritional interaction does not rely on biosynthesis of all B vitamins as originally suggested by Puchta [32]. For instance, similar to all the other *Arsenophonus* genomes, biosynthetic capacity for thiamin is compromised in *Ca.**Arsenophonus lipopteni*. The genome however possesses ABC thiamin transporter genes (*thiP, thiQ, tbpA*) implying the content of thiamin or thiamin pyrophosphate, compared to e.g. biotin or riboflavin, in the host blood might be sufficient for the insect metabolic needs (Novakova, unpublished data). Within the spectrum of known *Arsenophonus* genomes ranging from 0.57 Mb of *Ca.**Riesia pediculicola* to 3.5 Mb of *A. nasoniae*, representing various symbiotic types, the genomic sequence of *Ca.**Arsenophonus lipopteni* clearly reflects characteristics common for obligate mutualists. Furthermore, the phylogenetic reconstruction suggests an independent origin of this obligate association within Hippoboscidae.
